# Heart Performance Determination by Visualization in Larval Fishes: Influence of Alternative Models for Heart Shape and Volume

**DOI:** 10.3389/fphys.2017.00464

**Published:** 2017-07-04

**Authors:** Prescilla Perrichon, Martin Grosell, Warren W. Burggren

**Affiliations:** ^1^Developmental Integrative Biology Research Cluster, Department of Biological Sciences, University of North TexasDenton, TX, United States; ^2^Department of Marine Biology and Ecology, Rosenstiel School of Marine and Atmospheric Science, University of MiamiMiami, FL, United States

**Keywords:** mahi-mahi, red drum, larval fish, heart shape modeling, ventricular volume, stroke volume, cardiac output

## Abstract

Understanding cardiac function in developing larval fishes is crucial for assessing their physiological condition and overall health. Cardiac output measurements in transparent fish larvae and other vertebrates have long been made by analyzing videos of the beating heart, and modeling this structure using a conventional simple prolate spheroid shape model. However, the larval fish heart changes shape during early development and subsequent maturation, but no consideration has been made of the effect of different heart geometries on cardiac output estimation. The present study assessed the validity of three different heart models (the “standard” prolate spheroid model as well as a cylinder and cone tip + cylinder model) applied to digital images of complete cardiac cycles in larval mahi-mahi and red drum. The inherent error of each model was determined to allow for more precise calculation of stroke volume and cardiac output. The conventional prolate spheroid and cone tip + cylinder models yielded significantly different stroke volume values at 56 hpf in red drum and from 56 to 104 hpf in mahi. End-diastolic and stroke volumes modeled by just a simple cylinder shape were 30–50% higher compared to the conventional prolate spheroid. However, when these values of stroke volume multiplied by heart rate to calculate cardiac output, no significant differences between models emerged because of considerable variability in heart rate. Essentially, the conventional prolate spheroid shape model provides the simplest measurement with lowest variability of stroke volume and cardiac output. However, assessment of heart function—especially if stroke volume is the focus of the study—should consider larval heart shape, with different models being applied on a species-by-species and developmental stage-by-stage basis for best estimation of cardiac output.

## Introduction

The embryos and larvae of fishes are not simply small juveniles or adults, but rather transitional life forms that bridge the critical gap between the spawned egg and the sexually immature juvenile. Larval fishes, passing through stages of yolk sac, hatching, opening mouth and free-swimming, are particularly vulnerable to environmental stressors due to their small size, low energy reserves and limited migration capacities. All organs and their physiological regulation are established during these critical early phases of development. Therefore, a failure in organ development or homeostasis during larval stages could lead to detrimental consequences, if not death, for maturing fishes.

Among the most critical organ systems is the cardiovascular system, which is the first to function in the larval fishes (indeed, in any developing vertebrate). Ultimately, the teleost fish heart develops into a classically viewed arrangement of five distinct segments arranged in series: the sinus venosus, atrium, ventricle, conus, and bulbous arteriosus (Farrell and Jones, 1992; Icardo and Colvee, 2011; Burggren et al., in press). While substantial species-specific differences in details of cardiac form exist between adults of different fish species, many cellular and molecular processes that underlie especially early cardiac development are highly conserved in all vertebrates, and particularly across teleost fishes (Bakkers, 2011; Liu and Stainier, 2012). In fish embryonic stages, the heart clearly beats and generates a blood flow well before being clearly formed and structured (Icardo, 1996; Chico et al., 2008). Cardiomyocyte contractions are first observed when the linear heart tube is being formed and are mainly initiated at the venous pole. Contractions are at first uncoordinated peristaltic movements, later becoming regular and sequential between the emerging atrial and ventricular chambers (Bakkers, 2011). Valves are forming at this time, and each cardiac cycle is likely to include not only forward but also some retrograde flow until valve formation is complete. The forming heart must function before its development is complete in the adult configuration. The heart, responsible for blood convection, must respond to the rapidly changing demands of the developing larvae, including an initial role in angiogenesis followed by transport of respiratory gases (O_2_), nutrients (e.g., amino acids, carbohydrates, fats), cellular waste products, regulators (e.g., hormones, molecular chaperones), and pressure (arterial circulation) (Burggren, 2013; Burggren et al., in press).

Evaluations of anatomical configuration changes and cardiac rhythmicity (cardiac contraction) have been used as indicators of condition and health in larval fishes, but these variables do not tell the full story of cardiovascular performance. Stroke volume and ultimately cardiac output are critical for health and normal development (Burggren and Bagatto, 2008; Burggren et al., in press). Understanding the heart's mechanics is crucial for clinical or environmental research because many pathologies are strongly correlated with cardiac failure (Incardona et al., 2004, 2009; Nelson et al., 2016; Sørhus et al., 2016; Stieglitz et al., 2016). These impairments in cardiac function are often accompanied by accumulation of fluid (edema), as well as craniofacial and body defects (Incardona and Scholz, 2016; Sørhus et al., 2016). Physiological effects may appear as well, such as the decreased swimming performance in mahi-mahi (*Coryphaena hippurus*) associated with a reduction in cardiac contractility and blood ejection (Mager et al., 2014; Nelson et al., 2016; Stieglitz et al., 2016). No doubt, cardiac function is key factor to fish survival, and understanding how cardiac output develops in larval fishes will provide a powerful insight to physiological conditions of developing larvae as they face environmental challenges.

There is clear value in knowing cardiac output in larval fishes, but actually performing such measurements in early development can be quite challenging. First, the small physical scale renders impractical the use of conventional *in situ* physiological tools (e.g., various forms of flow meters, direct catheterization) designed for much larger animals. Second, development of larval fishes is highly dynamic, especially in early stages, so the incomplete structure and morphological changes of the cardiac chambers results in rapid changes in physiological performance and, consequently, how it should be measured. Thus, various imaging techniques (ultrasound, position emission tomography, magnetic resonance, video and confocal microscopy) have been used for visualizing and modeling the geometry and volume of the heart in different biological models (fish, human) (Park et al., 2005; Di Donato et al., 2006; Singleman and Holtzman, 2012). Among these numerous techniques, videomicroscopy, when properly employed, can be a simple, inexpensive method for measuring rapidly changing physiological parameters of *in vivo* larval heart. Videomicroscopy have been commonly used in various early life stages of bird, amphibian and fish models to rapidly calculate relative change in stroke volume for cardiac output estimation (Faber et al., 1974; Keller et al., 1990; Burggren and Fritsche, 1995; Hou and Burggren, 1995; Schönweger et al., 2000; Schwerte and Fritsche, 2003; Kopp et al., 2005; Bagatto and Burggren, 2006; Burggren and Blank, 2009; Khursigara et al., 2017). For larval fishes, *in vivo* imaging techniques that capture and analyze images of the intact, beating heart within the individual provide powerful tools for studying *in vivo* mechanisms (e.g., morphological, behavioral and molecular) in the context of physiologically realistic environments. *In vivo* imaging techniques provide numerous benefits, such as a non-invasive methodology with high-resolution images of the living animal in a potentially low stress situation, measurement repeatability over time, rapid data acquisition, and high reproducibility of quantitative data. Finally, these *in vivo* techniques provide successful and meaningful integrative tools to measure structures, functions and interactions of biological processes throughout an intact living organism with all its complexities, even in early developmental stages.

While videomicroscopy has been the tool of choice for analyzing cardiac function (e.g., stroke volume and calculation of cardiac output) in larval fishes, there are also frequently unappreciated pitfalls to be considered. The highly dynamic process of cardiogenesis, coupled with the fact that cardiac chambers display irregular, changing shapes through development prior to assuming the adult configuration, complicates cardiac output measurements, as does potential interspecific variation in heart dimension. Additionally, in the earliest stages, incomplete valve formation may result in retrograde flow during the cardiac cycle, which may not be accounted for by simple visualization of the heart. Despite these pitfalls, most cardiovascular studies measuring stroke volume in early life stages in fishes, as well as birds and amphibians, have modeled the heart as a simple rotary ellipsoid or prolate spheroid (see references above). To our knowledge, no studies have assessed the inherent error of stroke volume measurement that stems from employing this simple mode compared to other more elaborate and, perhaps more realistic, models of heart shape.

The aim of this study, then, was to assess the validity of three different heart models applied to both diastolic and systolic contraction events by determining inherent error and measurements artifacts, with the goal of providing guidance for better calculation of stroke volume and cardiac output. We have used cardiac image data previously acquired in developing larvae of mahi-mahi (hereafter mahi), a pelagic marine fish with rapid embryonic development, and the red drum (*Sciaenops ocellatus*). Shape and motion of the larval heart were tracked by digital imaging using conventional and novel modeling approaches, which were then compared for precision (repeatability, measurement with low variability).

## Materials and methods

### Image and video acquisition

**Figure 1:**
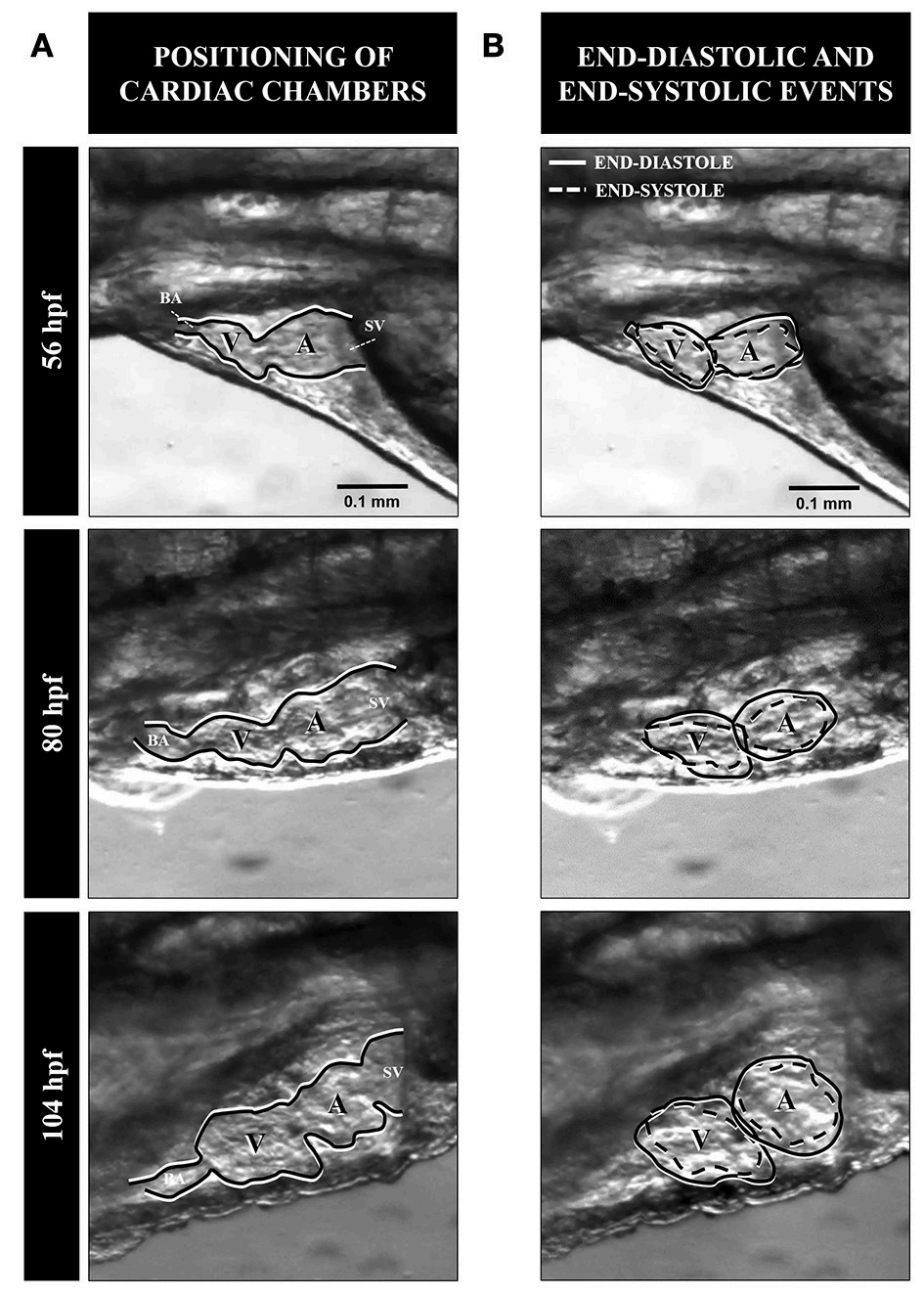
Shape and rhythmic changes of the heart of larval mahi during the early post-embryonic stages of 56, 80, and 104 h post-fertilization. **(A)** Morphology and general outline of cardiac chambers in left lateral view. **(B)** Heart chambers are outlined during end-diastole (solid line) and end-systole (dashed line). Note the relative changes in volume of the atrium and ventricle as development progresses. BA, Bulbous arteriosus; A, atrium; V, ventricle; SV, sinus venosus.

Video images were acquired from larval mahi and red drum in previous studies (Khursigara et al., 2017; Perrichon et al., 2017). All larval mahi and red drum used in those studies came from the Experimental Hatchery of the University of Miami (Florida) and the Texas Parks Wildlife–CCA Marine Development Center in Corpus Christi (Texas), respectively. Briefly, unanaesthetized larvae were individually immobilized in a Petri dish containing 2% methylcellulose/98% seawater and positioned on a thermally regulated microscope stage (Brook Industries, Lake Villa, IL) at 25°C (mahi) or 26°C (red drum). Temperatures corresponded to their respective rearing temperatures for broodstock after spawning. Larvae were then orientated in left lateral view for video capturing. Digital images of heart (Figure 1A) were recorded using videos camera Fire i400 (Unibrain, San Ramon, CA) mounted on Nikon SMZ800 stereomicroscope (Gx9.8). Twenty second-long live videos were digitized at 30 frames.s^−1^ using PhotoBooth and Nikon software and calibrated using a stage micrometer. A total of 36 red drum larvae at 56 h post fertilization (hpf) and 10 mahi larvae at 56, 80, and 104 hpf were used for calculation of ventricular variables in the current study.

### Comparison of ventricular shape models

#### Outlining of ventricular perimeter for stroke volume calculation

To assess the suitability of various models, morpho-volumetric measurements were made in fish larvae at 56 hpf in red drum and 56, 80, and 104 hpf in mahi—stages in which the cardiac chambers become functional and valve formation is completing. Previously published video images were processed by ImageJ software (Schneider et al., 2012). End-diastolic and end-systolic volumes of the ventricle were determined by outlining the ventricular perimeter (2D-circumference) (Figure 1B). This approach has been used previously in cardiac studies of other developing animals (Faber et al., 1974; Schönweger et al., 2000; Schwerte and Fritsche, 2003; Burggren et al., 2004; Kopp et al., 2005, 2010; Bagatto and Burggren, 2006).

**Figure 2:**
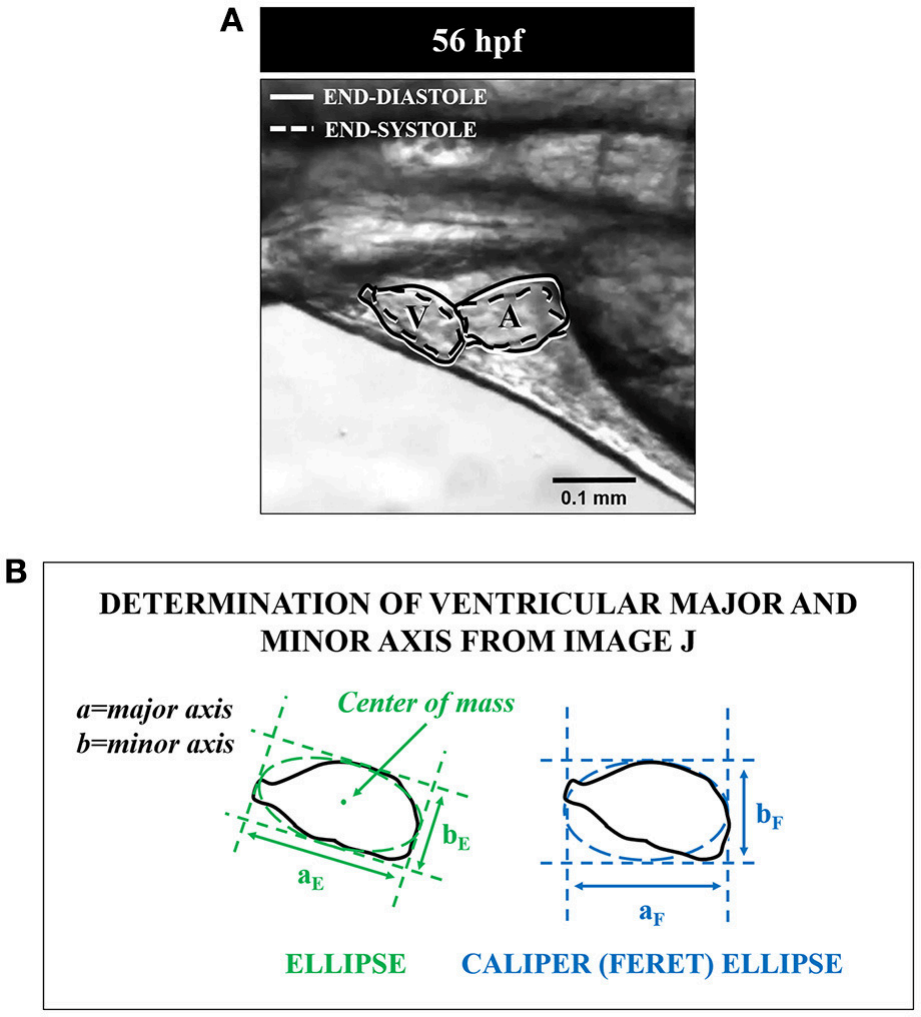
Determination of major and minor ventricular axes from two ellipsoid models established using ImageJ software in 56 hpf mahi. The **(A)** shows the actual images of the ventricle during end-diastole (solid line) and end-systole (dashed line). The **(B)** shows the different variables used in measurement of both a simple ellipse and a caliper ellipse. See text for further details. a_E_ and b_E_ represent minor and major axis for standard ellipse; a_F_ and b_F_ represent minor and major axis for caliper ellipse.

Based on the anatomical region of interest, the cardiac perimeter was then fitted with two different ellipses set up by ImageJ software, based on major and minor axes (Figure 2). First, ImageJ fitted the best ellipse based on the center of selection and extracted the primary and secondary axis (major and minor axis) from this fitted ellipse. Then, another form of ellipse was tested using caliper diameter (also known as Feret's diameter), which is the longest distance between any two points along the selection boundary and specified direction. Minimum and maximum caliper diameters were then extracted. Major and minor axis from both ellipse models were then exported and compared. Preliminary calculation showed no significant difference between respective minor (a_E_ and a_F_) and major (b_E_ and b_F_) axis of both caliper and standard ellipses (results not shown), so they have been averaged for volume calculations.

#### Sphericity and volumetric models

The first step in comparing the three models for cardiac shape was determing the ventricle's sphericity index, which is an indication of the “roundness” of an object (Di Donato et al., 2006). This parameter was calculated from the short- to long-axis ratio of the ventricle in both end-systole and end-diastole to identify is the degree of sphericity of the larval heart of mahi and red drum.

Precise measurements of stroke volume in larval mahi and red drum were then determined using three potential heart models: a prolate spheroid, (PS), a whole cylinder (CYL) and a pairing of a cone tip with a cylinder (80% of initial cylinder, CO-Tip + CYL). The volumes for each shape were calculated from the following equations:

$$\begin{array}{l} (1)\text{ Volume of a prolate spheroid},\;V_{PS}=\frac{\pi }{6}ab^{2}\\ (2)\text{ Volume of a cylinder},\;V_{CYL}=\frac{\pi }{4}ab^{2}\\ (3)\text{ Volume of a cone tip + cylinder},[V_{CO-Tip(20\%)}=\frac{\pi }{60}ab^{2}]\\ +\left[V_{CYL(80\%)}=\frac{\pi }{5}ab^{2}\right]\text{ equivalent to }V_{CO-Tip+CYL}=\frac{13}{60}\pi ab^{2} \end{array}$$

where *a* represents the major (longitudinal) axis and *b* is the minor (width) axis.

Three consecutive systolic and diastolic events for each larva were captured, analyzed and then averaged to provide a representative estimator of the cardiac cycle. Mean stroke volume (nL) was calculated as the difference between diastolic and systolic ventricular volumes. Finally, cardiac output for each model was calculated as the product of stroke volume and heart rate (from data shown in Perrichon et al., 2017).

### Statistical analyses

Statistical analyses were performed using Statistica 12 software. Shape index and volumetric variables were statistically evaluated with either one-way or two-way nested-ANOVA, followed by Tukey *post-hoc* test. Results are expressed as mean ± standard error of mean (SEM). A significance level of 5% was used for all analyses.

## Results

### Developmental changes in heart form

An important potential limitation for calculation of stroke volume of these both larval fishes is the highly dynamic structure and morphological changes of the cardiac chambers that occur literally hour to hour during early development (Figure 1A). This makes the typically adopted “one size fits all stages” approach to modeling heart volume inherently inaccurate. Specifically, between 56 and 80 hpf the developing heart is generally elongate for the species examined here. Importantly, the ventricle is initially smaller compared to the larger and nearly spherical posteriorly located atrium. At 56 hpf the anteriorly-located ventricle has a conical apical shape. However, heart form progressively changes with continuing development. From 104 hpf, the heart increasingly assumes its adult configuration in which the ventricle presents a more cylindrical-spheroid shape. The ventricle of mahi grow more rapidly than the atrium, and at 104 hpf the ventricle now equals or exceeds the size of atrium. Valves between the sinus venosus and atrium, and between the ventricle and bulbous arteriosus, are well differentiated at this stage.

A more spherical ventricle appears in later developmental stages, as evident from the sphericity index. This value is significantly lower (less spherical shape) at 56 hpf (0.45 ± 0.02 in end-diastole and 0.43 ± 0.02 in end-systole) and 80 hpf (0.42 ± 0.02 in end-diastole and 0.40 ± 0.01 in end-systole) compared to later in development at 104 hpf, irrespective of contraction state (0.54 ± 0.03 in end-diastole and 0.50 ± 0.03 in end-systole) (Table 1).

**Table 1:** Sphericity index of the ventricle during diastolic and systolic events through three post-embryonic stages in mahi.

		**Developmental stage**		
	**Contraction state**	**56 hpf**	**80 hpf**	**104 hpf**
Sphericity index	End-Diastole	0.45 ± 0.02^α^	0.42 ± 0.02^α^	0.54 ± 0.03^β^
	End-Systole	0.43 ± 0.02^α^	0.40 ± 0.01^α^	0.50 ± 0.03^β^

*Superscripted letters indicate significant differences through development (P < 0.05)*.

### Modeling stroke volume

Compared to the conventional prolate spheroid (PS) model, heart volume of mahi was calculated to be 50% higher using the CO-Tip + CYL model. This compares with a 30% larger volume using the simpler CYL model (Table 2). Based on the dimensional measurements of ventricular volumes in larval mahi (56 hpf), end-diastolic and stroke volumes were calculated to be 42 and 50% higher using the CYL heart model compared to the conventional PS one, respectively. Using video images of the red drum fish, end-diastolic and end-systolic volumes using CYL and CO-Tip + CYL models were significantly elevated by 50 and 30%, respectively, compared to the common PS model (Table 2). Stroke volumes in red drum were similar in both the CO-Tip + CYL (0.06 ± 0.01 nL) compared to the conventional PS model (0.05 ± 0.01 nL), but were 40% higher using the CYL model (0.07 ± 0.01 nL).

**Table 2:** Modeling of heart volume in larval mahi and red drum at 56 hpf.

**THEORETICAL MODEL**			**MEASUREMENTS FROM LARVAL FISHES (56 hpf)**		
**Shape models**		**Calculated volume (based on arbitrary values of *a* = 10 and *b* = 4)**	**Volume (nL)**	**Mahi**	**Red drum**
Prolate spheroid	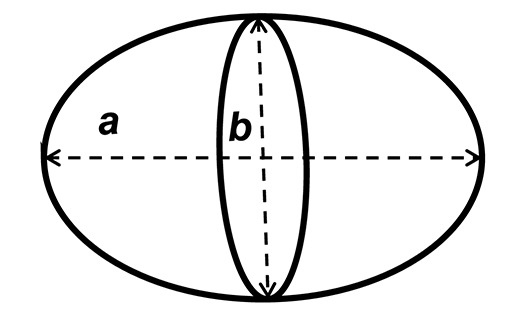	84	*V*_*DIA*_	0.31 ± 0.02^α^	0.19 ± 0.01^δ^
			*V*_*SYS*_	0.17 ± 0.02^α^	0.14 ± 0.01^δ^
			*V*_*SV*_	0.14 ± 0.01^α^	0.05 ± 0.01^δ^
Cylinder (100%)	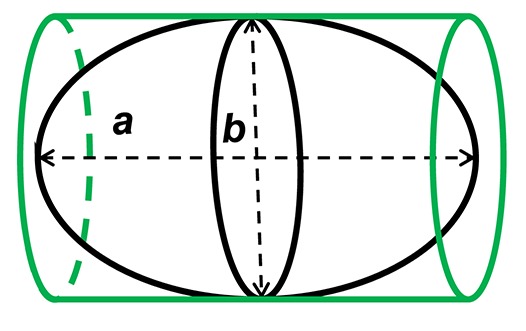	126	*V*_*DIA*_	0.44 ± 0.03^β^	0.28 ± 0.01^ε^
			*V*_*SYS*_	0.23 ± 0.02^α^	0.21 ± 0.01^ε^
			*V*_*SV*_	0.21 ± 0.02^β^	0.07 ± 0.01^ε^
Cone tip (20%) + cylinder (80%)	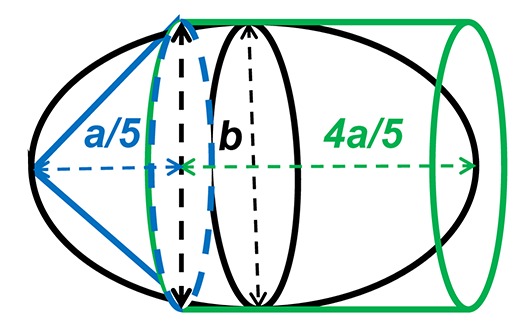	109	*V*_*DIA*_	0.38 ± 0.02^αβ^	0.24 ± 0.01^ε^
			*V*_*SYS*_	0.20 ± 0.02^α^	0.18 ± 0.01^ε^
			*V*_*SV*_	0.18 ± 0.02^αβ^	0.06 ± 0.01^δ^

*a: Major axis; b: Minor axis; VDIA: Diastolic Volume; VSYS: Systolic Volume and VSV: Stroke Volume*.

*Data presented as mean ± SEM. N = 10 and 36 in mahi and red drum, respectively. Superscripted letters indicate significant differences between models for a same volumetric variable in mahi (α; β) and red drum (δ; ε) respectively (P < 0.05)*.

A study of ventricular volume was made in mahi in post-embryonic stages at 56, 80, and 104 hpf to identify the calculation bias between different heart models (Table 3). Through the developmental range examined, end-systolic volumes remained statistically identical (*P* > 0.05) irrespective of heart model shape employed. At 56 and 80 hpf in mahi, end-diastolic and stroke volumes are significantly (*P* < 0.05) increased using CYL models compared to the common PS models. However, no significant differences were found for any heart shape model at 104 hpf, irrespective of the volumetric variables measured.

**Table 3:** Evaluation of diastolic, systolic and stroke volumes using three shape models through three post-embryonic stages (56, 80, and 104 hpf) of mahi.

			**MAHI LARVAE**		
**Shape models**		**Volume (nL)**	**56 hpf**	**80 hpf**	**104 hpf**
Prolate spheroid	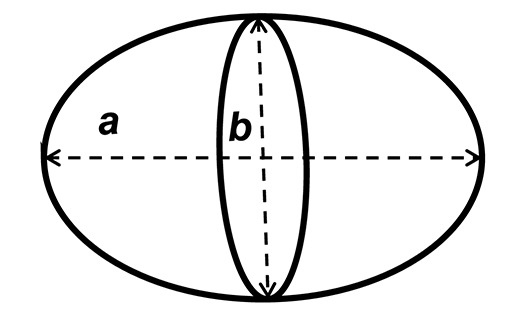	*V*_*DIA*_	0.31 ± 0.02^α^	0.40 ± 0.03^δ^	0.98 ± 0.12^κ^
		*V*_*SYS*_	0.17 ± 0.02^α^	0.26 ± 0.3^δ^	0.58 ± 0.07^κ^
		*V*_*SV*_	0.14 ± 0.01^α^	0.14 ± 0.02^δ^	0.40 ± 0.07^κ^
Cylinder (100%)	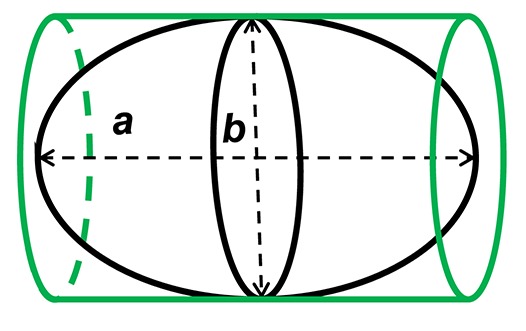	*V*_*DIA*_	0.44 ± 0.03^β^	0.54 ± 0.04^ε^	1.31 ± 0.17^κ^
		*V*_*SYS*_	0.23 ± 0.02^α^	0.32 ± 0.03^δ^	0.76 ± 0.10^κ^
		*V*_*SV*_	0.21 ± 0.02^β^	0.22 ± 0.02^ε^	0.54 ± 0.11^κ^
Cone tip (20%) + cylinder (80%)	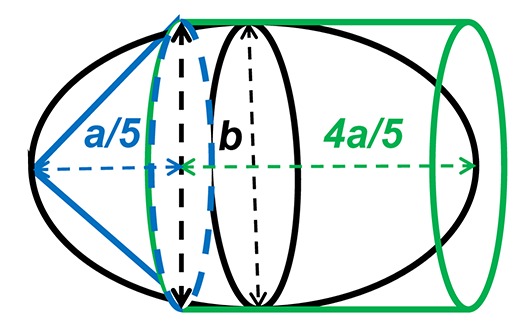	*V*_*DIA*_	0.38 ± 0.02^αβ^	0.47 ± 0.03^δε^	1.13 ± 0.15^κ^
		*V*_*SYS*_	0.20 ± 0.02^α^	0.28 ± 0.02^δε^	0.66 ± 0.09^κ^
		*V*_*SV*_	0.18 ± 0.02^αβ^	0.19 ± 0.02^δε^	0.47 ± 0.09^κ^

*a: Major axis; b: Minor axis; V_DIA_: Diastolic Volume; V_SYS_: Systolic Volume and V_SV_: Stroke Volume*.

*Data are Mean ± SEM. N = 10. Superscripted letters indicate significant differences between models at same developmental stages (P < 0.05)*.

### Calculating cardiac output

**Figure 3:**
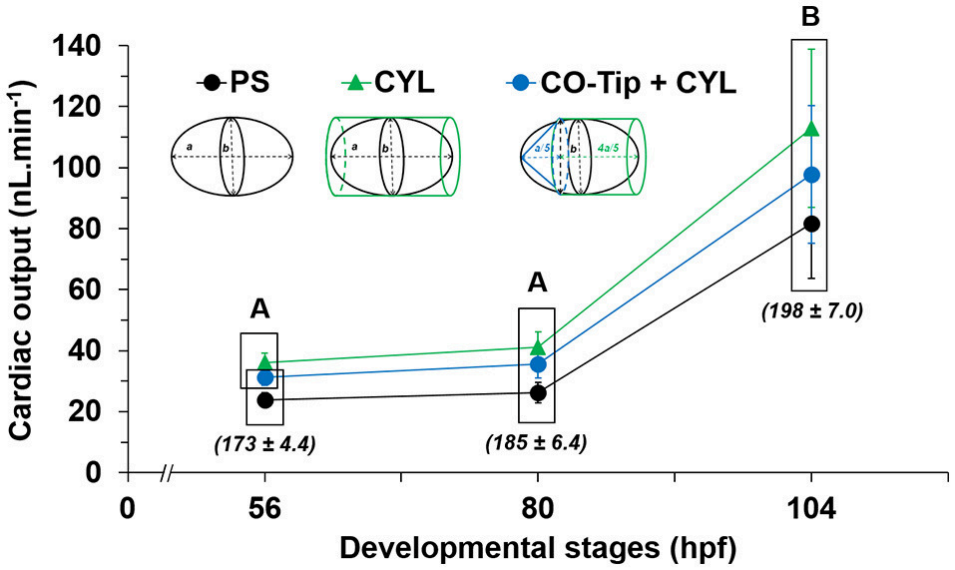
Cardiac output calculations as a function of development in larval mahi as determined by the three ventricular models: Prolate Spheroid, PS; Cylinder, CYL; and Cone Tip + Cylinder, CO-Tip + CYL. Heart rate (beats per minute) data are given in italic and in parentheses. *N* = 10 for each model at each developmental stage. Data are Mean ± SEM. A box surrounds statistically identical mean values at the same development time (*P* > 0.05). Capital letters indicate significant differences across development (*P* < 0.05).

Heart rate and stroke volume in each individual larva were multiplied to yield that larva's cardiac output. Cardiac output was relatively stable between 56 and 80 hpf (*P* > 0.05) but then increased significantly in stages (104 hpf) (*P* = 0.04 for PS; *P* < 0.01 for CO-Tip + CYL and CYL) (Figure 3). However, while stroke volume was relatively constant, there was much greater variation in individual heart rate. Consequently, there was no significant differences (*P* > 0.05) in cardiac output irrespective of the calculation models used.

## Discussion

This study has considered changing cardiac form during cardiogenesis to develop potentially more precise quantification of ventricular blood pumping in larval teleost fishes. Specifically, a common generic method of 2D fixed imaging for analyzing and characterizing the ventricular volumes in post-embryonic stages of the fishes mahi and red drum has been evaluated. Ventricular volumes were compared using three different models of heart shapes, taking into account the developmental changes in geometry of the ventricle (sphericity, conicity and cylindricity). We also determined inherent error of measurement artifacts for more precise calculation of stroke volume and cardiac output.

### Critique of videomicroscopy methodology

Previous studies employing videomicroscopy have frequently employed the “standard” prolate spheroid model in the measurement of stroke volume and calculation of cardiac output. Table 4 presents representative data using this model. Despite a taxonomic span from fishes to birds, there is similarity in the range of calculated values from different studies. Some of these intra- and interspecific differences could derive from that fact that clear delineation of diastole and systole using still photos of cardiac structures can be difficult because of the nature of heart motion. Some structures are at some points in the cardiac cycle covered by other structures and then at other points become clearly visible during the cardiac cycle. Viewing fixed image planes acquired by stereomicroscopy is unlikely to resolve this problem and outlining the ventricle to precisely measure ventricular volume may be imprecise using fixed images. Previous authors have recommended acquiring images of the ventricle from multiple angles to confirm ventricular outlines during the cardiac cycle (Bagatto and Burggren, 2006).

**Table 4:** Representative cardiovascular research in vertebrate embryos and larvae reporting stroke volume and cardiac output derived from prolate spheroid heart modeling.

**Species**	**Developmental stage**	**Body mass (mg)**	**Rearing conditions**	**Stroke volume (μL)**	**Cardiac output (μL^.^min^−1^)**	**References**
**BIRD**						
Chicken (*Gallus gallus*)	HH 16 to 25	Embryos: 13–115	T = 38°C, H = 50%	0.07–0.67^*^	12–110^*^	Faber et al., 1974
Chicken (*Gallus gallus*)	HH 16 to 24	Eggs: 55^.^10^3^–73^.^10^3^	T = 37.5°C, H = 55–65%	0.05–0.25	2–35	Burggren et al., 2004
**AMPHIBIAN**						
Anuran amphibian (*Xenopus laevis*)	NF 41 to 66	3–1^.^10^3^	T = 20–24°C	2.4^.^10^−3^–7.6	0.25–623	Hou and Burggren, 1995; Fritsche and Burggren, 1996
**FRESHWATER FISH**						
Rainbow trout (*Oncorhyncus mykiss*)	1 dph–150 atu	12–60	T = 5–15°C	1.8^.^10^−3^–0.1	0.2–20	Mirkovic and Rombough, 1998
Minnow (*Phoxinus phoxinus*)	42–480 hpf	Eggs: 3.3–3.7 Larvae:1.8–2.8	T = 10–25°C	1.1^.^10^−3^–3.0^.^10^−3^	125^.^10^−3^–410^.^10^−3^	Schönweger et al., 2000
Zebrafish (*Danio rerio*)	72–336 hpf	0.2–0.3	T = 25–31°C	0.10^.^10^−3^–0.50^.^10^−3^	40^.^10^−3^–100^.^10^−3^	Kopp et al., 2005, 2010
Zebrafish (*Danio rerio*)-	48–144 hpf	N/A	25°C	0.14^.^10^−3^–0.29^.^10^−3^	10^.^10^−3^–60^.^10^−3^	Bagatto and Burggren, 2006
**MARINE FISH**						
Red drum (*Sciaenops ocellatus*)	56 hpf	N/A	25°C	0.05^.^10^−3^	7^.^10^−3^	Khursigara et al., 2017
Mahi (*Coryphaena hippurus*)	32–128 hpf	N/A	25°C	0.07^.^10^−3^–0.36^.^10^−3^	9.5^.^10^−3^–64^.^10^−3^	Perrichon et al., 2017
Mahi (*Coryphaena hippurus*)	56 hpf	N/A	26 and 30°C	0.14^.^10^−3^	30^.^10^−3^	Perrichon et al., 2017

*atu: Accumulated thermal units; dph: days post-hatching; HH: Hamburger-Hamilton stage; hpf: hours post-fertilization; H: Humidity; NF: Nieuwkoop-Faber stage; T: temperature*.

* *Assumed values and calculated from the range of body mass given by authors*.

**Figure 4:**
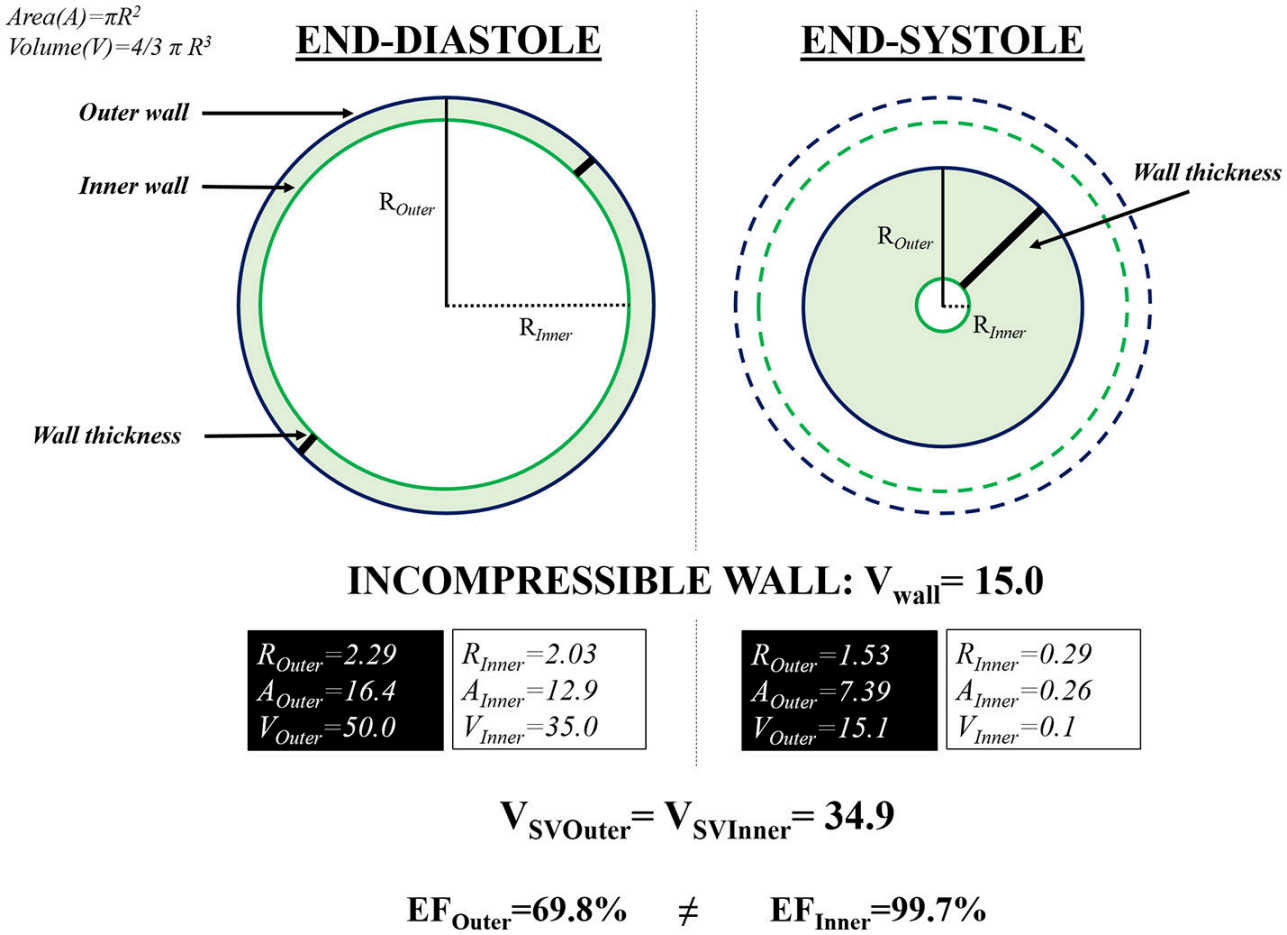
Simple schematic of the ventricle in cross-section, assuming that the ventricular wall can change shape (thickness) and that ventricular tissue is incompressible. Irrespective of whether the inner or outer ventricular wall is used for outlining, calculated stroke volume remains constant. A, area; EF, ejection fraction (%); R, radius or semi-diameter; V, volume; V_SV_, stroke volume. Adapted and modified from Stoylen (2001) and Stoylen et al. (1999, 2003).

There has also been discussion about what variables can be derived from the outline of the outer vs. the inner ventricular wall. This questions whether variation in ventricular wall thickness is a factor in accepting or rejecting videomicroscopy of the beating ventricle. We posit that, in theory, either outer or inner perimeter measurements are valid in calculating ventricular lumen areas and volumes, based on the important assumption that heart muscle is generally assumed to be incompressible. This means the total volume of this muscular wall remains constant irrespective of whether is stretched during diastole, for example. Figure 4 is adapted from extensive investigations of myocardial strain in the mammalian ventricle (Stoylen et al., 1999, 2003; Stoylen, 2001). Simultaneous transverse strains (expansion) of heart wall will result in calculation of a constant stroke volume irrespective of cyclic changes in the thickness of the *incompressible* ventricular wall. In other words, stroke volume will theoretically be identical whether measuring the inner or outer ventricular wall (Figure 4) based on this incompressibility. Past investigations of larval fishes, including the research of the current authors (Table 4) have opted for outline of the outer ventricular wall delineation, for the practical reason that videomicroscopy typically allows much clearer visualization of the outer than the inner ventricular wall.

Studies on stroke volume and cardiac output characterization in response to pharmaceutical substances, or simply in basic research of cardiac performance, have reported values of ejection fraction—the amount of blood pumped out of the ventricle at each contraction (Incardona et al., 2011). Ejection fraction is certainly useful for tracking changing heart performance (including failure). However, using videomicroscopy as deployed in this study does not permit precise calculation of ejection fraction for two reasons (Faber et al., 1974). First, the inner ventricle wall is, as already described, more difficult to precisely outline. Second, if the ventricle has an irregular inner surface with trabeculae and wall crypts, the overall inner ventricular outline would not precisely represent ventricular volume (Figure 4).

### Developmental changes in heart form

A changing geometry of the ventricle from initial cardiogenesis to adulthood occurs in developing fishes. For example in the zebrafish three main ventricular shapes have been recognized during different phases of development, depending on the video recording angle, degree of cardiac chambers looping and migration (Singleman and Holtzman, 2012). The earliest form is an ellipso-rectangular shape during advanced larval phases (3.4–12 mm), maturing into a triangular shape during juvenile and finally developing into a spherico-ellipsoid shape during the adult stage. While following a somewhat similar pattern of change in the larvae of other species, there appear also to be species-specific modifications in form of the developing heart in fishes. In mahi and red drum the ventricle is relatively elongate at 56 and 80 hpf of larval development (Incardona and Scholz, 2016; Figure 1). These species show an initial anterior conical apical shape, which then changes to a cylindro-spheroid shape without an apex by stage (104 hpf). This shape is then maintained during additional development. The progressive change of the ventricular geometry is reflected as well by an increasing sphericity index at 104 hpf, with the ventricle becoming more cylindrical or prolato-spherical (depending on the contraction/relaxation phase), in comparison to previous stages of development. These geometry changes are more important in end-diastole when the heart is dilated.

### Mathematical modeling stroke volume and cardiac output

Changes in geometric configurations of the mahi heart are closely tied to the maturation and growth of the whole fish. Regarding assessment of cardiac function, mahi exhibited a significant elevation in both stroke volume and cardiac output from 104 hpf, which is likely related to accelerating development and growth at this point in development (Perrichon et al., 2017). Similar patterns of increase in both stroke volume and cardiac output also occur in zebrafish and minnow larvae through early development (Schönweger et al., 2000; Bagatto and Burggren, 2006). Although visual measurement of cardiac output in embryos and larvae of aquatic organisms using a prolate spheroid is common place (Burggren and Fritsche, 1995; Hou and Burggren, 1995; Mirkovic and Rombough, 1998; Schönweger et al., 2000; Schwerte et al., 2003; Bagatto and Burggren, 2006), none of these prior studies considered alternative models using different shapes to calculate potential bias inferred by those models in the stroke volume calculation.

In the present study, statistically significant differences in diastolic volumes between the common prolate spheroid model (PS) and CO-Tip + CYL heart model occurred at 56 hpf in mahi. These differences between PS and Co-Tip + CYL models are also observed in both diastolic and systolic volumes in 56 hpf red drum and resulted in statistically different model-dependent values for stroke volume in both species (Table 2). These differences persisted at 80 hpf in mahi (Table 3). Generally, the common prolate spheroid model (PS) and CO-Tip + CYL model yielded statistically identical stroke volumes. However, the CYL model provided volumes 30–50% larger depending on the specific variable, which might bias the cardiac output calculation. Importantly, these variations in calculated values are closely tied to the geometric configuration of the developing heart. The CYL model does not take into consideration the anterior conical apex at 56 and 80 hpf. This is also highlighted at 104 hpf, where no statistical differences were found in volumes irrespective of model used. Regarding cardiac output, values were also higher at 56 hpf when the single cylindrical shape was used.

### Choosing an estimation model

With maturation in heart size and increasing heart sphericity, calculation of cardiac output may be influenced by choice of heart model. In mahi, calculated cardiac outputs using the different models were statistically identical at 56, 80, and 104 hpf, despite significant differences in calculated ventricle volume. Of course, an additional variable in any cardiac output calculation is heart rate, which can be quite variable in larval fishes (Schönweger et al., 2000; Bagatto and Burggren, 2006; Perrichon et al., 2017; Burggren et al., in press). In the present study, the heart rate variability was sufficiently large (± 4.4 to 7 beats.min^−1^ from 56 to 104 hpf) eliminate any statistical difference in calculated cardiac output between models, despite statistically significant differences in calculated stroke volume (Figure 3).

Determination of the best model to most precisely estimate the ventricular stroke volume and subsequently calculate cardiac output in developing fishes is quite difficult and depends of various parameters. Calculation of stroke volume or cardiac output is based on both ventricular geometry (and subsequent stroke volume calculation) and heart rate, as mentioned above. Certainly, the conventional, most frequently used prolate spheroid model appears to be the easiest to employ, requiring the simplest calculations. Using similar image analysis techniques, reproducible results for cardiac output have been generated in amphibian larvae (Orlando and Pinder, 1995; Fritsche and Burggren, 1996; Schwerte et al., 2003) and several freshwater fishes, such as the zebrafish, minnow and rainbow trout (Mirkovic and Rombough, 1998; Schönweger et al., 2000; Kopp et al., 2005, 2010; Bagatto and Burggren, 2006). However, employment of different models for calculation of stroke volume should be considered when the morphology of the ventricle of larval fishes (or other animals) differs markedly between species. This is especially true if the goal is to look at stroke volume, perhaps as an indicator of the underlying cardiac mechanics, rather than overall cardiac output. With low heart rate variation, quite large differences in cardiac output calculations might emerge at any given developmental stage, and consideration of different models at different stages might more clearly reveal the developmental changes in cardiac performance (Tables 2, 3).

Finally, it is important to note that no technology that we are aware of allows us to cross-validate measurements of cardiac output in larval fishes, which often weight <1 mg. This raises consideration of “precision” vs. “accuracy.” Obviously, being precise and accurate is the most desirable condition. However, depending on the purpose of the research in question, relative yet precise changes in stroke volumes or cardiac output between specific experimental conditions (e.g., different temperatures, oxygen levels or toxicant levels) can be highly valuable and robust. For example, a recent study on larval red drum using ventricular assessment with videomicroscopy has revealed significant differences in cardiac output between control populations and those exposed to pollutants derived from petroleum (Khursigara et al., 2017). Thus, irrespective of the actually accuracy of cardiac output measurement, the precision of the methodology allows investigation of various stressors, as well as normal developmental effects, on cardiovascular physiology.

## Conclusions

Videomicroscopy for quantification of cardiac output in larval fishes is effective, relatively easy to use, inexpensive and non-invasive. Notably, however, differences in shape and dimension of the larval ventricle exist both between species and within species as a function of development when the prolate spheroid model is applied, especially when employing outer ventricular wall dimensions. No method is valid for determination of ejection fraction, however. Models other than the prolate spheroid might be applied, however, when marked differences in ventricular shape present themselves, or when assessment of stroke volume *per se* is required. Irrespective of model employed, videomicroscopy of the beating, *in vivo* ventricle provides a powerful tool to explore and understand the impact of pharmaceutical substances or environmental conditions (e.g., pollutants, treatments, temperature, hypoxia, UV) on the physiological fitness of developing larval fishes. Finally, future methods should be developed considering measurements of blood pressure/speed to better determinate the accuracy in the measurement system of stroke volume.

## Ethics statement

All handling and use of animals in the present study were authorized by the Institutional Animal Care and Use Committees (IACUC) of the University of Miami (Mahi-mahi, No 15-019 and 15-067) and the University of Texas Austin (Red drum, No AUP-2014-00375).

## Author contributions

PP collected the original data in a previous study, and developed the first draft of the manuscript. WB edited and wrote new sections for the manuscript and refined some of the model calculations. MG secured funding for the reported studies, provided edits for the manuscript and provided facilities and infrastructure for measurements on mahi larvae.

### Conflict of interest statement

The authors declare that the research was conducted in the absence of any commercial or financial relationships that could be construed as a potential conflict of interest.
